# Characterisation of APS-1 Experimental Models Is Crucial for Development of Novel Therapies

**DOI:** 10.1155/2023/7960443

**Published:** 2023-01-11

**Authors:** Sarah Almaghrabi, Thomas Lovewell, Mimoun Azzouz, Rachid Tazi Ahnini

**Affiliations:** ^1^Department of Infection, Immunity and Cardiovascular Disease, Sheffield, UK; ^2^Sheffield Institute for Translational Neuroscience (SITRaN), Department of Neuroscience, The Medical School, University of Sheffield, Sheffield S10 2RX, UK

## Abstract

Autoimmune polyglandular syndrome type 1 (APS-1) is an inherited autosomal disorder. The most common clinical features of the disease include adrenocortical failure, hypoparathyroidism (HP), and chronic mucocutaneous candidiasis (CMC). APS-1 is caused by mutations in the autoimmune regulator (AIRE) gene. AIRE is a transcriptional factor involved in the regulation of thousands of genes in the thymus. It facilitates central tolerance by promoting the ectopic expression of tissue-specific antigens (TSAs) in medullary thymic epithelial cells (mTECs), leading to the deletion of self-reactive thymocytes. Several Aire-deficient mice were developed separately, on different backgrounds; seven published Aire knockout mice show a variety of phenotypes depending on the strain used to generate the experimental model. The first Aire-deficient mice were generated on a “black 6” background almost 20 years ago. The model showed mild phenotype with relatively modest penetrance compared to models generated on BALBc or NOD backgrounds. The generation of all these experimental models is crucial for development and testing new therapeutics as well as reading the response to treatments.

## 1. Introduction

Autoimmune polyglandular syndrome type 1 (APS-1), also known as autoimmune polyendocrinopathy candidiasis ectodermal dystrophy (APECED), is a monogenic autosomal recessive disorder [1–4] and a rare devastating primary immunodeficiency disease (PID) as previously reviewed [5–7]. Linkage studies have indicated that the autoimmune regulator (*AIRE*) is responsible for the pathogenesis of APS-1 and is caused by loss-of-function mutations in *AIRE* [8–10], resulting in a dysfunctional AIRE protein. *AIRE* represents a single gene defect resulting in a multisystem autoimmune disease. AIRE is a transcriptional regulator that is highly expressed in medullary thymic epithelial cells (mTECs) [11, 12]. One of the pivotal findings is that AIRE/Aire promotes self-tolerance in the thymus by regulating the promiscuous expression of a wide array of expressions of tissue-specific antigens (TSAs) that have the commonality of being tissue-restricted in their expression pattern in the periphery [13, 14]. Autoreactive thymocytes (T cell precursors) that recognise these TSAs with high affinity undergo negative selection via apoptosis, or, alternatively, forkhead box P3 (FOXP3)^+^ regulatory T cells (T_reg_) are generated in order to prevent autoimmunity [15]. Researchers rapidly cloned the *Aire* mouse, to develop an experimental model of APS-1 [16, 17]. Studies using *in vivo* Aire^−/−^ mouse models have significantly elucidated and increased the knowledge of APS-1 autoimmunity [14]. These findings highlight the importance of central tolerance in controlling autoimmunity. In fact, the clinical picture is highly variable with great phenotypic heterogeneity, even among siblings [18]. This notion was further supported by studies in Aire^−/−^ mice that demonstrated differing patterns of tissue-specific autoantibodies depending on the background mouse strain, suggesting that individual major histocompatibility complex might influence the disease phenotype [19]. *In vivo* murine model studies have led to a significant increase in the knowledge of the mechanisms involved in the autoimmunity of APS-1 patients [14, 20]. Several Aire-deficient mice, on different backgrounds, have been developed separately; Table 1 summarises the seven published strains generated by four different research groups [16, 17, 19, 21, 22]. Here, we have analysed the expression of Aire-regulated genes and lymphocytic infiltration of tissues in both presymptomatic and postsymptomatic Aire-deficient mice on a C57BL/6 background. Phenotypically, all strains look grossly normal and resemble wild-type littermates. However, closer investigations revealed spontaneous lymphocytic infiltration towards multiple tissues, circulating autoantibodies and infertility that increases with age, except for the mice generated by Matsumoto's group [21, 23]; they are fertile but produce offspring only occasionally. In order to investigate the effect of genetic variations on the variability between human APS-1 patients, Jiang et al. backcrossed C57BL/6J, NOD/LtJ, BALB/cJ, and SJL/J background mice to *Aire^−/−^* mice generated previously by [17] [19]. In agreement with their previous study, Aire-deficient mice exhibited lymphocytic infiltrates and autoimmune endocrine destruction with slight differences in the set of pathologies developed between the different strains. Furthermore, their study confirmed that variation of genetic background affects the overall intensity of the disease, which ranges from mild in C57BL/6J background to infiltration that is more aggressive; pneumonotis and high autoantibodies production were detected in NOD/LtJ background mice [19]. Conflicting evidence has been debated as to whether *Aire^−/−^* mice are a relevant model for the human APS-1 disease [24, 25]. Pontynen et al. [24] concluded that the *Aire^−/−^* mouse model failed to develop the same autoantibody profile as APS-1 patients. These observations were made in the genetic background of C57BL/6, which generates milder immune phenotype than other strains such as NOD mice (Table 2) and as previously reviewed [5–7]. Immunologically, the *Aire^−/−^* phenotype is characterised by the presence of multiple autoantibodies against endocrine and nonendocrine organs, similar to APS-1 patients. For this very reason, *Aire^−/−^* can mimic APS-1 phenotype and can be considered as an adequate model for this disease. Here, we analysed Aire deficiency on C57BL/6 background in presymptomatic and postsymptomatic phases, which could shed light into the design and development of future clinical trials in patients with APS-1.

## 2. Materials and Methods

### 2.1. Breeding and Genotyping of Transgenic Mice

For all *in vivo* studies, B6.129S2-*Aire^tm1.1Doi^*/J (The Jackson Laboratory stock 004743) were used. The homozygous *Aire^−/−^* mice lack the murine Aire protein. All mice were maintained in a controlled facility in a 12-hour dark-light photocycle with free access to food and water. All *in vivo* experimental work was approved by the local ethics committee and performed in accordance with the UK Home Office Animals (Scientific Procedures) Act 1986.

To create this mutant, the originating investigator employed a targeting vector bearing a loxP site-flanked Pgk-neo cassette inserted into intron 2. An additional single loxP site was placed in intron 1. The targeting construct was electroporated into 129S2/SvPas-derived D3 embryonic stem (ES) cells. Correctly targeted ES cells were injected into C57BL/6 blastocysts. The resulting chimeric mice were mated with CMV-cre transgenic mice on a C57BL/6 background. Exon 2 and portions of introns 1 and 3 were excised in the offspring. The resulting chimeric animals were crossed to C57BL/6 mice. Heterozygous offspring were crossed to C57BL/6 for at least nine generations.

Heterozygous *Aire^−/−^* mice were used for breeding, with litters genotyped at 2 weeks after birth by PCR, using the KAPA Mouse Genotyping Kit (KR0385, KAPA Biosystems). Briefly, DNA was extracted from ear clips in 100 *μ*l solution containing 10X KAPA Express Extract Buffer, 1 U/*μ*l KAPA Express Extract Enzyme, and PCR-grade water for 10 minutes at 75°C and inactivated at 95°C for 5 minutes. Three primers were used, recognising *Aire* with two different reverse primers specific for either the wild-type gene or the mutant gene (Table 3).

The PCR reaction consisted of 1 *μ*l of DNA template, 0.5 *μ*M of each of the forward primer, wild-type and mutant reverse primers, 12.5 *μ*l of 2X KAPA2G Fast (HotStart) Genotyping Mix with dye, and H_2_O to a final volume of 25 *μ*l. The PCR product was electrophoresed on a 3% agarose gel with 0.5 *μ*g/*μ*l ethidium bromide at 120 V for 60 minutes. Wild-type animals generated one band at 19 bp, homozygous transgenic animals generated one band at 140 bp, and heterozygous carriers generated two bands, one at 195 bp and one at 140 bp.

### 2.2. Tissue Collection

Mice were euthanized by intraperitoneal injection of 500 mg/kg of sodium pentobarbital (sodium pentobarbital, 20% *w*/*v* solution for injections, JML). Tissues were collected and immediately fixed by 4% paraformaldehyde (4% PFA) overnight at 4°C. After 24 hours, the tissues were transferred to PBS. Tissues were paraffin embedded and sectioned at 5 *μ*m. To analyse AAV9-GFP transduction efficiency, thymi were embedded in OCT and frozen at -80°C. After sectioning, 1 in 10 slides was taken throughout the thymus for analysis. This equated to 15 thymic sections analysed per animal.

### 2.3. Immunofluorescence

For Aire staining in APS-1 mouse model thymus, sections were incubated in xylene twice for 10 minutes before being hydrated through 100%, 95%, and 70% ethanol. First, they were washed in H_2_O for 1 minute and then in PBS for 5 minutes and then incubated with a serum-free protein block buffer (DAKO), for 10 minutes at room temperature. Subsequently, they were incubated with 0.3% Triton X-100 in PBS, for 10 minutes (for membrane permeabilization) and then further incubated with a polyclonal goat antibody against AIRE D-17 (sc-17986, Santa Cruz Biotechnology), at a dilution of 1 : 50 in 0.15% Triton X-100 in PBS, for 1 hour at room temperature, or overnight at 4°C. Lastly, they were washed three times, for 10 minutes each time, with PBS only, before a secondary antibody was added. The secondary antibody used was Alexa Fluor 488 donkey anti-goat IgG antibody (Invitrogen); this was diluted 1 : 500 in PBS and incubated at room temperature for 1 hour before being washed in PBS three times for 10 minutes. For GFP visualisation in AAV9-GFP thymic sections, slides were fixed with acetone for 20 minutes. All slides were mounted using VECTASHIELD Anti-fade Mounting Medium with DAPI (Vector Labs) and allowed to be cured overnight at room temperature. Images were taken with a fluorescence microscope (Leica AF6000) and were analysed using ImageJ software. Confocal microscope images were taken using Nikon A1 confocal microscope system.

### 2.4. RNA Extraction and Quantitative PCR

#### 2.4.1. RNA Extraction

Total RNA was extracted using the TRI Reagent® (Sigma-Aldrich). Tissues were homogenised using mortar and pestle with liquid nitrogen. TRI reagent was added on the powdered tissues and transferred into 1.5 ml Eppendorf tubes for subsequent phase separation. The aqueous phase containing the RNA was separated and transferred to a fresh tube, and then, the RNA was precipitated by adding 0.5 ml of isopropanol per 1 ml of TRI reagent. Pelleted RNA was then washed with 75% ethanol and resuspended in 30 *μ*l of RNase-free water. RNA concentration was measured by NanoDrop. RNA was reverse-transcribed, and cDNA was synthesized using SuperScript® IV First-Strand Synthesis System kit by random hexamer (18091050, Invitrogen) according to the manufacturer's protocol.

#### 2.4.2. Real-Time Quantitative PCR

Samples were used for quantitative RT-PCR (qPCR). QPCR reaction consisted of 10 *μ*l power SYBR™ Green PCR Master Mix (4367659, Applied Biosystems), cDNA, and 600 nM forward and reverse primers (Table 4) to a total volume of 20 *μ*l, which was run in triplicate. The PCR reaction was carried out in 384-well plate using 7900HT real-time PCR system (Applied Biosystems); PCR cycling conditions were 95°C for 10 minutes for one cycle, 95°C for 30 seconds, and 60°C for 60 seconds for 40 cycles.

To explore the effect of Aire on downstream genes, a subset of Aire-dependent and Aire-independent genes, thymi were collected from *Aire^+/+^* and *Aire^−/−^* littermates for thymic *ex vivo* mRNA measurements. Three groups of TSA genes were selected: Aire-dependent upregulated genes, Aire-dependent downregulated genes, and Aire-independent genes (Table 4). These TSAs were selected based on their expression levels in C57BL/6 mice at 3-4 weeks old, Affymetrix Mouse Genome 430 2.0 data NCBI GEO accession no. GSE8564 as described by [26].

#### 2.4.3. Statistical Analysis

Data were analysed using two-way ANOVA and Tukey's multiple comparison test (*P* < 0.05).

## 3. Results

Male and female mice of both *Aire^−/−^* and *Aire^+/+^* genotypes were examined at two different ages, presymptomatic (4-week-old, *n* = 8) and at the first signs of disease appearance (12-week-old, *n* = 8), using histological and molecular techniques.

**Figure 1:**
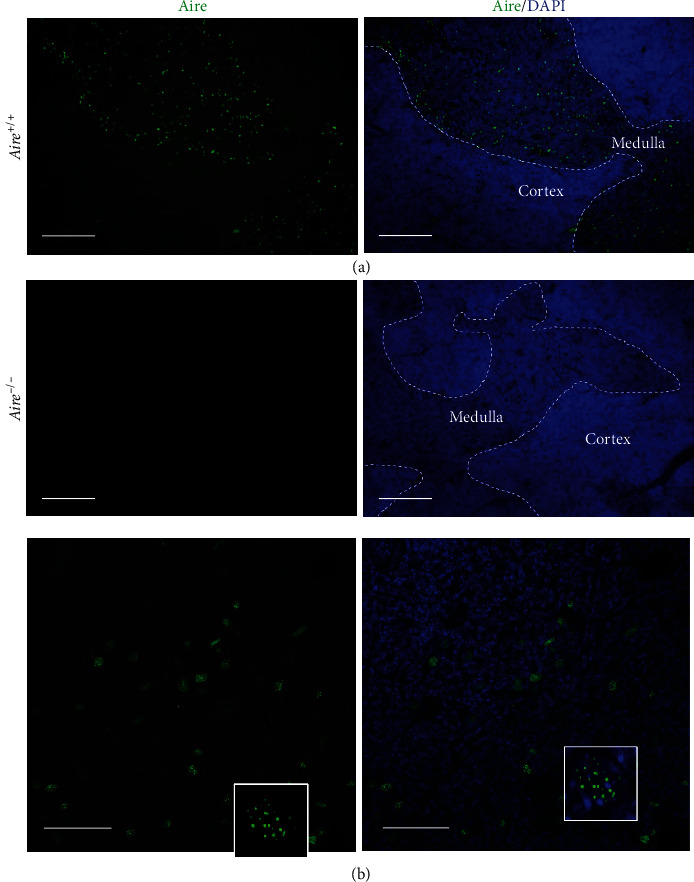
Aire expression in the thymus. Thymic sections from *Aire^+/+^* and *Aire^−/−^* mice. The sections labelled with anti-Aire antibody and nuclei counterstained with DAPI (a, b). (a) Thymic section from wild-type mouse (*Aire^+/+^*). (b) Thymic section from homozygous Aire knockout mouse (*Aire^−/−^*). Scale bar 200 *μ*m. (c) Higher magnification (scale bar 50 *μ*m) showing the subcellular localisation of Aire. Thymic sections from *Aire^+/+^* mice show that Aire is expressed in the nucleus in a punctate pattern within a subset of mTECs.

B6.129S2-*Aire^tm1.1Doi^*/J mice, a well-stablished Aire^−/−^ mice [17], were purchased from the Jackson Laboratory (USA). The Benoist and Mathis group engineered this APS-1 mouse model to carry a defective Aire gene that represents the most common APS-1 mutation, R257X, by introducing lox/Cre-mediated recombination in embryonic stem (ES) cells. These mice carry, in the homozygous state, a mutant allele bearing a deletion of exon 2 and portions of the upstream and downstream introns [13]. To confirm the expression pattern of Aire in the thymus of *Aire^+/+^* and *Aire^−/−^* mice, thymi were collected from 4-week-old and 12-week-old mice for histological analysis. Using immunofluorescence (IF) staining on 4-week-old *Aire^+/+^* (*n* = 3) and *Aire^−/−^* (*n* = 3) thymic sections, a subset of mTECs were found to be positive for Aire in *Aire^+/+^* but not *Aire^−/−^* mice (Figure 1). For subcellular location, Aire is localised to the nucleus in distinct punctate nuclear dot structures within a subset of mTECs, confirming previous data [27–29].

**Figure 2:**
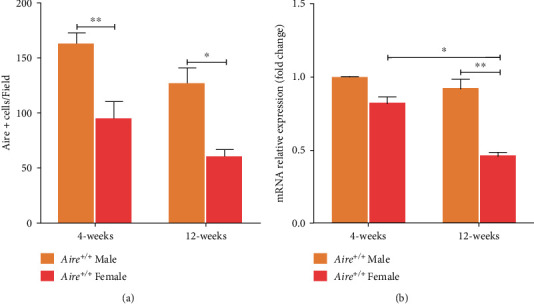
Aire expression in *Aire^+/+^* thymi in males and females mice. (a) Semiquantification of Aire+ cells in thymic sections from 4-week- and 12-week-old *Aire^+/+^* mice. Random fields per thymic section were counted, *n* = 5 per thymic section microscopic field (×20) for males and females, *n* = 2 mice per group. (b) *Aire* gene expression in thymi from 4-week- and 12-week-old males and females. Data were analysed using two-way ANOVA and Tukey's multiple comparison test (*P* < 0.05). Error bars represent ±SEM.

To assess Aire protein expression levels, we performed IF staining of thymic sections from APS-1 mice. Aire-positive cells (Aire+) in the thymic sections of APS-1 mice were counted to compare the expression levels of Aire between males and females and to assess the expression levels in two age groups: 4-week-old (*n* = 4) and 12-week-old mice (*n* = 4). Randomly chosen, nonoverlapping, 5 fields per thymic section per mouse were counted for Aire+ cells at ×200 magnification. The mean number of Aire+ cells in 4-week-old *Aire^+/+^* mice was 163 cells for male thymic sections and 95 cells for female thymic sections. Thus, male thymic sections showed a significantly higher Aire+ cells compared to females (*P* = 0.0022). In 12-week-old *Aire^+/+^* mice, thymic sections showed a slight reduction in Aire+ cells in both male and female. The mean number of Aire+ cells in 12-week-old *Aire^+/+^* male mouse thymic sections was 127 cells and 60 cells for 12-week-old *Aire^+/+^* female mouse thymic sections. Although 12-week-old *Aire^+/+^* male mouse thymic sections exhibited lower numbers of Aire+ cells than 4-week-old *Aire^+/+^* male mouse thymic sections, this difference was not significant. On the other hand, the number of Aire+ cells in 12-week-old *Aire^+/+^* female mouse thymic sections was significantly lower when compared to 4-week-old *Aire^+/+^* female mouse thymic sections (*P* = 0.0438) (Figure 2(a)).

*Aire* expression at the mRNA level was determined by qPCR and normalised to beta actin (*β*-actin), a housekeeping gene. *Aire* mRNA was expressed at higher levels in male thymi when compared to female thymi in both age groups (Figure 2(b)). In 4-week-old *Aire^+/+^* mice, this was not significant. However, in 12-week-old *Aire^+/+^* mice, *Aire* expression levels were significantly higher in males compared to females (*P* = 0.0049). In addition, *Aire^+/+^* female mice displayed significantly lower *Aire* mRNA levels at 12 weeks old when compared to those at 4 weeks old (*P* = 0.0121). However, for *Aire^+/+^* male mice, *Aire* expression levels were not significantly different between the two age groups.

### 3.1. Expression Profile of Aire-Dependent and Aire-Independent Genes

To explore the effect of Aire on downstream genes, a subset of Aire-dependent and Aire-independent genes were chosen to be investigated. Thymi were collected from *Aire^+/+^* (*n* = 2) and *Aire^−/−^* (*n* = 2) littermates for thymic *ex vivo* mRNA measurements. Three groups of TSA genes were selected: Aire-dependent upregulated genes, Aire-dependent downregulated genes, and Aire-independent genes (see Table 5 in the method section). These TSAs were selected based on their expression levels in C57BL/6 mice at 3-4 weeks old, Affymetrix Mouse Genome 430 2.0 data (NCBI GEO accession no. GSE8564) [26].

**Figure 3:**
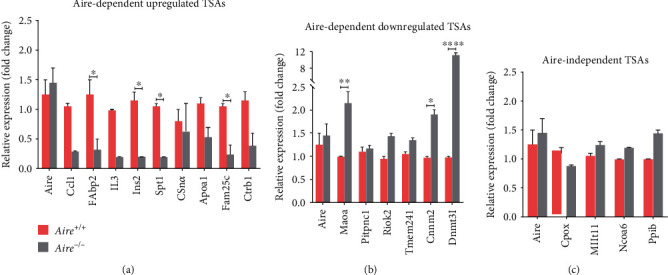
TSA expression pattern in *Aire*^+/+^ and *Aire*^−/−^ mice. Quantitative PCR analysis of expression of 15 Aire-dependent TSAs and 5 Aire-independent TSAs in thymic cells obtained from 4-week-old *Aire*^+/+^ mice (*n* = 2) and *Aire*^−/−^ mice (*n* = 2). Results normalised to the expression of *β-actin* and presented relative to the expression values in *Aire^−/−^* thymic cells. Data were analysed by two-way ANOVA and Sidak's multiple comparison test. ^∗∗∗∗^*P* < 0.0001, ^∗∗∗^*P* < 0.0005, and ^∗∗^*P* < 0.005. ± SEM.

In this part of the study, qPCR results showed that the 9 Aire-dependent upregulated TSAs exhibited higher expression levels in *Aire^+/+^* mice and the 6 Aire-dependent downregulated TSAs exhibited higher expression levels in *Aire^−/−^* mice. The absence of Aire does significantly influence Aire-dependent groups in each group. For all nine Aire-dependent upregulated genes tested, a demonstrably substantial decrease (3-14-fold change) in expression levels was observed for 4-week-old *Aire^−/−^* mice compared to *Aire^+/+^* mice. *Fam25c* displayed a significant 14-fold reduction in *Aire^−/−^* when compared to its level of expression in *Aire^+/+^* mice (*P* = 0.0454). *CCL1* showed a decline by almost 4-fold in *Aire^−/−^* compared to its expression level in *Aire^+/+^* mice. *Ins2* and *Spt1* expression levels significantly decreased by 5-fold (*P* = 0.0133 and *P* = 0.0317, respectively). *IL3* exhibited the same pattern but was not considered significant. Moreover, *Csnα*, *Ctrb1*, and *Fabp2* expression levels dropped by 7-fold because of Aire depletion. *Apoa1*, a principal protein component of high-density lipoprotein (HDL), was reduced by almost 3-fold in *Aire^−/−^* mice (Figure 3).

For Aire-dependent downregulated TSA genes, *Dnmt3I*, an epigenetic factor that contributes to the establishment of DNA methylation and histone modification [30], showed significantly high expression levels in *Aire^−/−^* mice with almost a 12-fold change when compared to its expression levels in *Aire^+/+^* mice (*P* = <0.0001). *Maoa*, a key regulator for normal brain function [31, 32], and *Cnnm2*, an important player in magnesium homeostasis [33], were expressed significantly higher in *Aire^−/−^* than *Aire^+/+^* mice with a 2.5-fold increase (*P* = 0.0097) and 2-fold increase (*P* = 0.0446), respectively. *Pitpnc1*, *Riok2*, and *Tmem241* expression levels in *Aire^−/−^* mice were increased by almost 1.3-fold when compared to their expression levels in *Aire^+/+^* mice.

The analysis of Aire-independent TSA genes revealed that with the presence or absence of Aire, their level of expression in the thymus behaved in an Aire-independent manner [26]. *Cpox*, *Mllt11*, *Ncoa6*, and *Ppib* changed in expression levels ranged between 0.85- and 1.38-fold in *Aire^−/−^* mice when compared to *Aire^+/+^* mice and were not significantly different.

### 3.2. Pathology in Aire-Deficient Mice

Phenotypically, the *Aire^−/−^* mice did not differ from their *Aire^+/+^* littermates. Therefore, tissue histology was investigated in 4-week-old and 12-week-old *Aire^−/−^* and *Aire^+/+^* mice with special attention given to the retina, lung, liver, stomach, and reproductive organs based on previous studies [12, 13].

**Figure 4:**
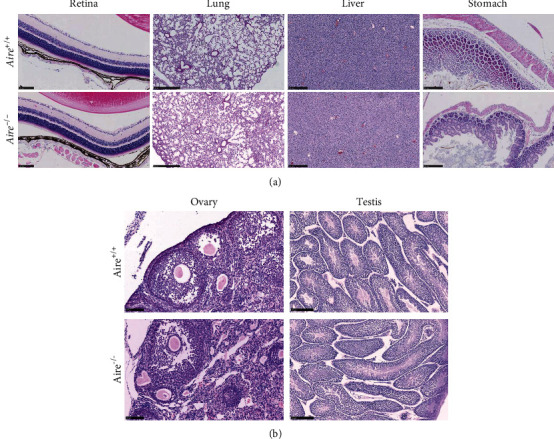
(a) Representative tissue sections from 4-week-old *Aire^+/+^* mice and *Aire^−/−^* mice. Retina, lung, liver, and stomach sections from *Aire^+/+^* (upper lane) and *Aire^−/−^* (lower lane) stained with haematoxylin and eosin (H&E). Scale bar: retina 100 *μ*m, lung 500 *μ*m, and liver and stomach 250 *μ*m. (b) Representative tissue sections from 4-week-old *Aire^+/+^* mice and *Aire^−/−^* mice. Ovary and testis sections from *Aire^+/+^* (upper lane) and *Aire^−/−^* (lower lane) stained with haematoxylin and eosin (H&E). Scale bar: ovary 100 *μ*m and testis 250 *μ*m.

In 4-week-old mice, no observable lymphocytic infiltration was detected in either the retina, lung, liver, and stomach (Figure 4(a)) or ovary and testis (Figure 4(b)). Thus, consistent with the previous studies, there was not any difference between *Aire^−/−^* mice and their *Aire^+/+^* littermates at 4 weeks old [12, 13].

**Figure 5:**
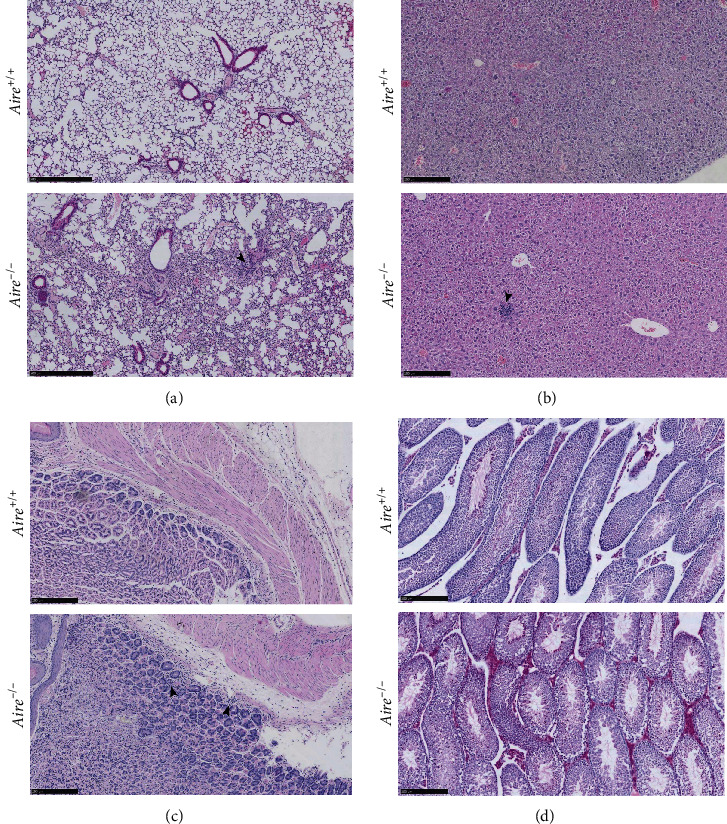
(a) Representative tissue sections from the lung of 12-week-old *Aire^+/+^* mice and *Aire^−/−^* mice. Lung sections from *Aire^+/+^* (upper lane) and *Aire^−/−^* (lower lane) stained with haematoxylin and eosin (H&E). Lymphocytic infiltration in *Aire^−/−^* lung (arrowhead). Scale bar 500 *μ*m. (b) Representative tissue sections from the liver of 12-week-old *Aire^+/+^* mice and *Aire^−/−^* mice. Liver sections from *Aire^+/+^* (upper lane) and *Aire^−/−^* (lower lane) mice stained with haematoxylin and eosin (H&E). Lymphocytic infiltration in *Aire^−/−^* mouse liver (arrowhead). Scale bar 250 *μ*m. (c) Representative tissue sections from the stomach of 12-week-old *Aire^+/+^* and *Aire^−/−^* mice. Stomach sections from *Aire^+/+^* (upper lane) and *Aire^−/−^* (lower lane) mice stained with haematoxylin and eosin (H&E). Lymphocytic infiltration in *Aire^−/−^* mouse stomach (arrowhead). Scale bar 250 *μ*m. (d) Representative tissue sections from the testis of 12-week-old *Aire^+/+^* mice and *Aire^−/−^* mice. Testis sections from *Aire^+/+^* (upper lane) and *Aire^−/−^* (lower lane) mice stained with haematoxylin and eosin (H&E). Leydig cell hyperplasia (LCH) like morphology in *Aire^−/−^* mouse testis. Scale bar 250 *μ*m.

**Figure 6:**
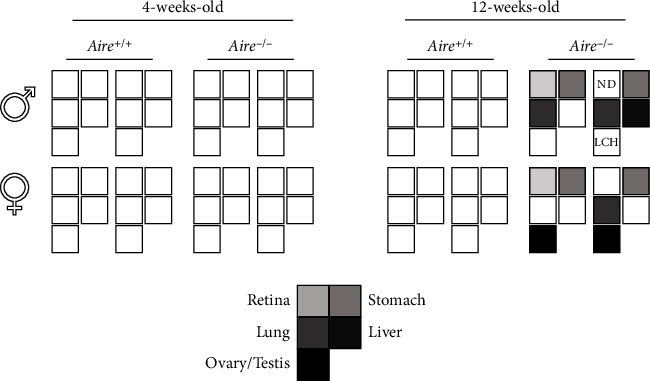
Summary of tissue infiltrate in *Aire^+/+^* mice and *Aire^−/−^* mice. In 4-week-old mice, no tissue infiltrations were observed in both *Aire^+/+^* and *Aire^−/−^* mice. In 12-week-old mice, tissues with lymphocytic infiltration highlighted for each *Aire^−/−^* mouse.

In the 12-week-old *Aire^+/+^* mice, no observable infiltrations were detected in their tissues. However, all four 12-week-old *Aire^−/−^* mice exhibited lymphocytic infiltration in some, but not all, investigated organs. For instance, moderate lymphocyte infiltration was detected in the lungs (Figure 5(a)), with mild lymphocytic infiltration detected in the liver and stomach (Figures 5(b) and 5(c)). Moreover, the testis of one 12-week-old *Aire^−/−^* mouse exhibited a Leydig cell hyperplasia (LCH) morphology suggestive of autoimmune gonadal atrophy (Figure 5(d)). A summary of tissue infiltration in *Aire^+/+^* and *Aire^−/−^* mice at 4 weeks and 12 weeks old is shown in Figure 6.

## 4. Discussion

The characterisation of Aire^−/−^ models is a crucial step towards understanding the link of AIRE to autoimmunity as recent genome-wide association studies find AIRE as a risk gene for pernicious type I diabetes, autoimmune Addison's disease, and anemia, respectively [34–36]. In this study, we demonstrated that Aire displays a sexually dimorphic expression pattern, with more elevated expression in males than females. There were more Aire-positive cells in male thymi than for females and higher relative mRNA expression levels as previously observed [37]. Another study demonstrated that oestrogen in females upregulates the number of methylated CpG sites in *Aire* promoter, leading to reduced Aire expression in mTEC [38]. This association between Aire and oestrogen may partially increase female susceptibility to autoimmune diseases. Furthermore, we show here that Aire expression levels, at both mRNA and protein levels, decrease with age, with significant reduction observed in 12-week-old *Aire^+/+^* females. Coder et al. found that this gradual age-dependent decline in Aire expression has been found to be due to the reduction in the frequency of Aire+ mTECs, but not due to the reduction of total mTECs [39]. We recently reported that intrathymic injection of an AAV9-AIRE therapeutic restored AIRE expression in the thymus of *Aire^−/−^* mice. This AIRE gene delivery led to a significant increase in TSA expression and reduced serum autoantibodies in treated mice [40]. Thus, the level of Aire expression per mTECs and Aire+ mTECs frequency is both critical for maintaining self-tolerance.

Aire regulates the transcription of a large set of TSA in thymic mTECs [13]. To confirm the Aire-dependent TSA expression profile in *Aire^+/+^* and *Aire^−/−^* mice, qPCR analysis was performed on thymic extracts to measure and compare TSA mRNA levels. In total, 19 TSAs were screened to determine their level of expression in both *Aire^+/+^* and *Aire^−/−^* mice at 4 weeks old. These were divided into three groups: Aire-dependent upregulated TSAs, Aire-dependent downregulated TSAs, and Aire-independent TSAs. Our data confirms that there was a consistent pattern of expression in each group. In the absence of Aire, the expression of Aire-dependent upregulated TSAs was dramatically reduced, whereas Aire-dependent downregulated TSAs were moderately induced. On the other hand, Aire-independent TSAs exhibited a neutral expression pattern. This screening will give an indication on the most affected genes in the absence of functional Aire and provides a baseline of the TSA gene expression levels in 4-week-old *Aire^−/−^* mice.

Another hallmark of Aire-deficient mice that was investigated in this study is the presence of lymphocytic infiltration of multiple tissues. In this part of the study, various tissues were investigated including the skin, brain, eye, heart, lung, liver, stomach, spleen, pancreas, kidney, testis, and ovary. Lymphocyte infiltration was previously observed in some of these tissues in *Aire^−/−^* mice on C57BL/6 background at 12 weeks old, when the first signs of disease appear [12]. For instance, we observed lymphocytes in the lung, liver, and stomach of 12-week-old *Aire^−/−^* mice, which are common infiltrations reported in Aire-deficient mice on C57BL/6 background [12, 13]. Moreover, LCH morphology has been observed in one 12-week-old *Aire^−/−^* male mouse; this could be an effect of hypogonadism which is associated with spermatogenic failure. Tazi et al. reported that Leydig cell hyperplasia manifests in the adult by signs of hypogonadism [41]. This finding suggests a gonadal atrophy comparable to APS-1 patients. There were no obvious pathological changes in other organs from the 12-week-old *Aire^−/−^* mice examined in this study. In contrast, no such changes were observed in 12-week-old *Aire^+/+^* mice. In agreement with the previous studies, the infiltrations showed a clear age dependence pattern, as tissue infiltration in 4-week-old *Aire^−/−^* mice was undetectable [12, 13].

Our data suggests that Aire-deficient mice on C57BL/6 background are asymptomatic at 4 weeks old and start to show the first signs of pathological (but not morphological) changes at 12 weeks old. These include gene expression of Aire-regulated genes as well as first tissue infiltration targeting organs such as the lung, liver, stomach, and testis. This is a crucial knowledge for the development of therapies. In fact, we have recently shown that direct injection of *AIRE* into the thymus of Aire-deficient mice was able to rescue the wild-type phenotype in 4-week-old mice but not in 12-week-old mice [40].

Together, this study analysed in details Aire^−/−^ mouse on C57BL/6 background; such model could be useful for future therapeutic studies.

## Figures and Tables

**Table 1:** APS-1 mouse models.

Background	Peltonen group	Mathis group				Matsumoto group		Scott group
	C57BL/6	C57BL/6	NOD/LtJ	BALB/cJ	SJL/J	C57BL/6	NOD/Shi Jic	C57BL/6
Design	Neo cassette insertion in exon 6	Cre-lox-mediated deletion of exon 2	Backcrossed with *Aire^−/−^* C57BL/6	Backcrossed with *Aire^−/−^* C57BL/6	Backcrossed with *Aire^−/−^* C57BL/6	Neo cassette insertion replacing exons 5-12	Backcrossed with *Aire^−/−^* C57BL/6	Cre-lox-mediated deletion of exon 8

Predicted mRNA	Exon 1-5	Exon 1				Exons 1-4		Exons 1-7

Predicted protein domains	HSR, NLS, premature SAND	Premature HSR				HSR, NLS		HSR, NLS, SAND, premature PHD-1

Major human equivalent mutation	C889T	R257X				NA		d1094-1106 del13

References	[16]	[17, 19]				[21, 23]		[22]

**Table 2:** Summary of *Aire^−/−^* mice affected systems.

Background	Peltonen group	Mathis group				Matsumoto group		Scott group
	C57BL/6	C57BL/6	NOD/LtJ	BALB/cJ	SJL/J	C57BL/6	BALB/c	C57BL/6
Digestive system	—	✓	✓	✓	✓	—	✓	—
Respiratory system	—	✓	✓	✓	✓	—	—	—
Eye/vision	—	✓	✓	✓	—	—	—	✓
Endocrine/exocrine glands	✓	✓	✓	✓	—	✓	✓	✓
Liver/biliary system	✓	—	✓	✓	✓	—	—	—
Reproductive system	✓	✓	✓	✓	✓	—	—	✓
Mortality	—	—	✓	—	—	—	—	—

**Table 3:** Primers used for genotyping.

Primer	Sequence 5′-3′	Concentration
Aire wild-type reverse	GGAGACTTGCCTATTCCTGTC	0.5 *μ*M
Aire mutant reverse	CCGGCGGATTTGTCCTAC	0.5 *μ*M
Aire forward	AGACTAGGTGTTCCCTCCCAACCTCAG	0.5 *μ*M

**Table 4:** Q-PCR primer sequence.

cDNA	UCSC code	Primer sequence	Location within cDNA	Size (bp)
Aire	uc007fww.2	For 5′CAGCAACTCTGGCCTCAAAG 3′ Rev 5′CTTCGAACTTGTTGGGTGTATAA 3′	518-802	285
AIRE	uc062arj.1	For 5′AGGCAACAGTCCAGGAGGTG 3′ Rev 5′TAGGGGTTCCCCAGGTGGAC 3′	1755-1885	131
Ccl1	uc007kmu.1	For 5′GGCTGCCGTGTGGATACAG 3′ Rev 5′AGGTGATTTTGAACCCACGTTT 3′	107-325	219
Fabp2	uc008rex.2	For 5′GTGGAAAGTAGACCGGAACGA 3′ Rev 5′CCATCCTGTGTGATTGTCAGTT 3′	359-475	117
IL-3	uc007ixn.1	For 5′GGGATACCCACCGTTTAACCA 3′ Rev 5′AGGTTTACTCTCCGAAAGCTCTT 3′	124-262	139
Ins2	uc009kog.3	For 5′GCTTCTTCTACACACCCATGTC 3′ Rev 5′AGCACTGATCTACAATGCCAC 3′	231-377	147
Spt1	uc029svn.1	For 5′CTGGTGAAAATACTGGCTCTGAA 3′ Rev 5′AGCAGTGTTGGTATCATCAGTG 3′	178-293	116
Csn*α*	uc029viy.2	For 5′ACCTTACTCCCAAAGCTGTCCTTA 3′ Rev 5′GAGGGTCCAGTCACATCAAATGT 3′	870-1005	136
Apoa1	uc009phb.3	For 5′ GGCACGTATGGCAGCAAGAT 3′ Rev 5′ CCAAGGAGGAGGATTCAAACTG 3′	182-310	129
Fam25c	uc007tat.1	For 5′GAGCAGTTCACGCAGTGGAA 3′ Rev 5′GCATGGGTAACAGCATCAGTG 3′	103-274	172
Ctrb1	uc009nms.2	For 5′ATGGCATTCCTTTGGCTTGTG 3′ Rev 5′GGATAGCATCCTCTCCGTTGAC 3′	19-142	124
Maoa	uc009ssa.2	For 5′GCCCAGTATCACAGGCCAC 3′ Rev 5′CGGGCTTCCAGAACCAAGA 3′	161-277	117
Pitpnc1	uc011ygq.1	For 5′CAACCCATCATGTGCTCCTAC 3′ Rev 5′CCCGAACATCATCCATTGTCAT 3′	1310-1484	175
Riok2	uc008ape.2	For 5′TAAGCTGTTCAACAATCCCTCC 3′ Rev 5′GCTGCTTGGTAAACACATTGG 3′	1730-1855	126
Tmem241	uc008ebx.2	For 5′TCTGCACCTGTTACCTGGCT 3′ Rev 5′AATGTCTGCCACCCTTGGAAT 3′	120-216	97
Cnnm2	uc008hue.1	For 5′AAGTGGCCCACCGTGAAAG 3′ Rev 5′CGCTTCTACTTCTGTTGCTAGG 3′	1951-2078	128
Cpox	uc007zoa.2	For 5′ACGGGCGTGTGTTTGAAAAG 3′ Rev 5′CACAGAACTTACACCCATAGCAG 3′	774-925	152
MIIt11	uc033hxa.1	For 5′TAAGTAGCCAGTACAGCTCCTT 3′ Rev 5′CGTAGGTAGGTGTATCTGACAGG 3′	14-115	102
Ncoa6	uc008nkm.2	For 5′GAATGTGCCCAACTTGTTACAC 3′ Rev 5′CCCTTCAATCTGAACGGAGAGAA 3′	353-536	184
Ppib	uc009qei.1	For 5′GGCTCCGTCGTCTTCCTTTT 3′ Rev 5′ACTCGTCCTACAGATTCATCTCC 3′	155-276	122
Fezf2	uc007sfs.2	For 5′ACTCGGCCTTGACAGCTGAACG 3′ Rev 5′TGAGCATTGAACACCTTGCCGCAC 3′	1063-1183	121
Foxn1	uc007kjc.2	For 5′TTCCATCAGTACTCCCCGGGTGG 3′ Rev 5′GCGTTGGCCTGGGGTGCAAT 3′	831-925	95
*β*-Actin	uc009ajk.2	For 5′GGCTGTATTCCCCTCCATCG 3′ Rev 5′CCAGTTGGTAACAATGCCATGT 3′	193-346	154

**Table 5:** List of Aire-dependent and Aire-independent genes.

Pattern of expression	Genes
Aire-dependent upregulated	Chemokine (C-C motif) ligand 1 (CCL1)
	Fatty acid-binding protein 2 (Fabp2)
	Interleukin 3 (IL-3)
	Insulin II (Ins2)
	Salivary protein 1 (Spt1)
	Casein *α* (Csn*α*)
	Apolipoprotein A-I (Apoa1)
	Family with sequence similarity 25 C (Fam25c)
	Chymotrypsinogen B1 (Ctrb1)

Aire-dependent downregulated	DNA methyltransfer-3-like (Dnmt3I)
	Monoamine oxidase A (Maoa)
	Phosphatidylinositol transfer protein cytoplasmic 1 (Pitpnc1)
	RIO kinase 2 (Riok2)
	Transmembrane protein 241 (Tmem241)
	Cyclin M2 (Cnnm2)

Aire-independent	Coproporphyrinogen oxidase (Cpox)
	Mixed lineage leukemia translocated to 11 (Mllt11)
	Nuclear receptor coactivator 6 (Ncoa6)
	Peptidylprolyl isomerase B (Ppib)

## Data Availability

Data is available on request. The authors can also make data available on request through the authors themselves.
